# Morphology and molecular evidence support the validity of *Pogonias courbina* (Lacepède, 1803) (Teleostei: Sciaenidae), with a redescription and neotype designation

**DOI:** 10.1371/journal.pone.0216280

**Published:** 2019-06-19

**Authors:** María de las Mercedes Azpelicueta, Sergio Matías Delpiani, Alberto Luis Cione, Claudio Oliveira, Alexandre Pires Marceniuk, Juan Martín Díaz de Astarloa

**Affiliations:** 1 CONICET, División Zoología Vertebrados, Museo de La Plata, Facultad de Ciencias Naturales, La Plata, provincia de Buenos Aires, Argentina; 2 CONICET, Laboratorio iBOL de referencia de Mar del Plata, Instituto de Investigaciones Marinas y Costeras, Facultad de Ciencias Exactas y Naturales, Mar del Plata, provincia de Buenos Aires, Argentina; 3 CONICET, División Paleontología, Museo de La Plata, Facultad de Ciencias Naturales, La Plata, provincia de Buenos Aires, Argentina; 4 Departamento de Morfologia Instituto de Biociências, UNESP, São Paulo, Brazil; 5 CONICET, Laboratorio de Biotaxonomía Morfólogica y Molecular de Peces, Instituto de Investigaciones Marinas y Costeras, Facultad de Ciencias Exactas y Naturales, Mar del Plata, provincia de Buenos Aires, Argentina; Pontificia Universidade Catolica do Rio Grande do Sul, BRAZIL

## Abstract

The family Sciaenidae comprises about 300 species. The black drum *Pogonias cromis* was the only valid species of the genus. Herein, *Pogonias courbina* Lacepède 1803 is redescribed based on morphological and molecular evidence and a neotype is designated. *Pogonias courbina* is distinguished by the following characters: the occurrence of characteristic thickening of the dorsal spines VII to XI in all specimens larger than 250 mm SL; all pterygiophores in the dorsal-fin laminar, thin; anal-fin pterygiophores slender excluded those of spines; lateral projections of gas bladder with few finger-like projections; genetic distance between both species 1%; exclusive occurrence of characters in six informative sites of COI (58 G; 214 G; 328 A; 331 A; 553C; 580 G). The method Automatic Barcode gap Discovery detected gaps in nucleotid distance congruent with the NJ, MP, and ML tree analysis. Also, advertisement calls are three times shorter in duration in *P*. *courbina* than in *P*. *cromis*. In addition, two monophyletic groups for *P*. *cromis* and *P*. *courbina* appear in trees obtained with different methodologies, emphasizing the absence of shared haplotypes. A gap of about 8000 km occurs in the distribution of both species along coastal areas of the Atlantic Ocean.

## Introduction

Members of the family Sciaenidae, currently assigned to Order Acanthuriformes, are widespread fish species. They live in tropical and temperate, marine, and brackish environments, and in freshwater in South America [1]. This large family (nearly 300 recognized species) includes commercially important and game fishes as the Red Drum, Black Drum, weakfish, sea trouts, kingfishes, white seabass, corvinas, roncadoras and the endangered Mexican Totoaba and Chinese Bahaba [2]. Among many different common names, they are called croakers and drums in English, pescadas and castanhas in Portuguese, and corvinas or pescadillas in Spanish. The vernacular names croakers, drums, or even “roncadoras” come from the sound they produce. Species of *Pogonias* (black drums in English, miraguaias in Portuguese, corvinas negras in Spanish) are demersal and estuarine fishes, distributed along coastal waters in both the North West and South West Atlantic Ocean [3, 4]. *Pogonias cromis* (Linnaeus, 1776) was the only valid species of the genus, distributed along the coast from the State of Massachusetts to the Gulf of Mexico and from the State of Rio de Janeiro to south of Buenos Aires Province in Argentina [4]. A large tropical gap determines an antitropical distribution between populations from southeastern North America and southern eastern South America. Two fossil species based on otoliths were described as *Pogonias styriacus* Weinfurter 1952 from Austria and *P*. *stringeri* Takeuchi and Huddleston 2008 from the Miocene of California [5, 6]. In this paper, the fishes from northern and southern Western Atlantic were confirmed as pertaining to two different species of *Pogonias*, *P*. *cromis* and *P*. *courbina* Lacepède 1803, an available name for southern populations [7]. Based on strong morphological and molecular evidence the redescription of *P*. *courbina* is provided and the designation of a neotype is included, adding information on physiological and biogeographical evidence.

## Material and methods

A total of 104 specimens of *Pogonias cromis* and *P*. *courbina* was examined based on the examination of both external (morphometrics and meristics) and internal (osteological) characters. Some skeletons were prepared by removing soft tissue. Radiographs were also taken, especially from those specimens preserved in fish collections and some fresh specimens. In addition, tissue samples have been collected from fresh specimens for molecular analysis. All large specimens were collected by artisanal or commercial fisheries from different regions of Western Atlantic Ocean in North and South America. Small and medium size specimens belong to different collections. Fish specimens were obtained from commercial and artisanal fisheries, and were not sacrificed for the present study.

Twenty one morphometric characters were measured on the left side of individuals to the nearest 0.1 mm using dial calipers. Measurements are expressed either as percents of standard length (SL) or percents of head length (HL), or otherwise indicated, and are defined as follows: Total length (1)–measured as a straight line from anteriormost end of upper lip to posteriormost tip of caudal fin. Standard length (2)–measured as a straight line from anteriormost end of upper lip to caudal-fin base (posterior end of hypural plate). Head (3) and snout lengths (4)–measured from anteriormost end of upper lip to posterior end of opercular bone and to anterior fleshy margin of eye, respectively. Eye diameter (5)–measured as greatest fleshy diameter of eye. Post ocular length (6)–measured from posterior margin of eye to first dorsal-fin ray. Predorsal length (7)–measured from anteriormost end of upper lip to base of first fin ray in dorsal fin. Prepelvic length (8)–measured from anteriormost point of upper lip to base of first fin ray in the pelvic fin. Upper jaw length (9)–measured from anterior margin of upper lip to posterior end of maxilla. Maximum body depth (10)–measured from base of first dorsal-fin ray to base of first pelvic-fin ray. Pectoral-fin length (11)–measured as length of longest fin ray. First dorsal-fin depth (12)–measured from base of first dorsal-fin ray to end of longest dorsal-fin ray. First dorsal-fin base length (13)–measured from base of first fin ray to base of last fin ray. Second dorsal-fin depth (14)–measured from base of first fin ray to end of longest dorsal-fin ray. Second dorsal-fin base length (15)–measured from base of first fin ray to base of last fin ray. Caudal-peduncle depth (16)–measured as least depth across caudal peduncle. Pelvic-fin length (17)–measured as length of longest fin ray. Pelvic-fin origin/anal-fin origin (18). Anal-fin base (19)–measured from base of first fin ray to base of last fin ray. Anal-fin depth (20)–measured from base of first fin ray to the end of the longest anal-fin ray. Interorbital width (21)–measured as width between the orbits.

Measurements 1 to 6 were taken between parallel lines while measurements 7 to 21 are straight-line distances. Meristic data were also taken from the left side of each specimen. Meristic values followed by an asterisk correspond to those of the holotype. Numbers of perforated lateral-line scales were counted from the scale above the pectoral-fin base to that located at the base of the caudal-fin rays. Counts of oblique rows of scales were made from the lateral line to bases of the dorsal and pelvic-fin rays. Vertebral numbers were obtained either from dissection or from cleared and stained specimens, or were taken from radiographs of type and non-type specimens. The first ural centrum was included as one vertebra.

### Nomenclatural act

The electronic edition of this article conforms to the requirements of the amended International Code of Zoological Nomenclature, and hence the new names contained herein are available under that Code from the electronic edition of this article. This published work and the nomenclatural acts it contains have been registered in ZooBank, the online registration system for the ICZN. The LSID is urn:lsid:zoobank.org:pub: 165AFCD5-4A13-48F1-B2AE-5E555F432008. The ZooBankLSIDs (Life Science Identifiers) can be resolved and the associated information viewed through any standard web browser by appending the LSID to the prefix "http://zoobank.org/". The electronic edition of this work was published in a journal with an ISSN, and has been archived and is available from the following digital repositories: PubMed Central and LOCKSS.

### Institutional acronyms

ANSP: Academy of Natural Sciences of Philadelphia, Drexel University, Philadelphia; AZUSC: Acervo Zoologico da Universidade Santa Cecilia, Santos, São Paulo; MACN: Museo Argentino de Ciencias Naturales Bernardino Rivadavia, CONICET, Buenos Aires; MLP: Museo de La Plata, Universidad de La Plata, La Plata; MNHN: Museu national d'histoire naturelle, Paris; INIDEP: Instituto Nacional de Investigaciones desarrollo pesquero, Mar del Plata; FLMNH: Florida Museum of Natural History, University of Florida, Gainsville; LBP: Laboratório de Biologia e Genética de Peixes, Universidade Estadual Paulista, Botucatu, São Paulo; UNMDP: Universidad Nacional de Mar del Plata; USNM: National Museum of Natural History, Smithsonian Institution, Washington.

### Extraction, PCR amplification and DNA sequencing

DNA was extracted from the muscle tissue of each specimen using an automated glass Fiber method [8]. The 650 bp barcode region of COI was subsequently amplified under the following DNA barcoding protocols and primer cocktails developed for fish [9]. Specimen information and barcode sequence data from this study were compiled using the Barcode of Life Data Systems [10]. The sequence data is publicly accessible on BOLD and GenBank.

The DNA sequences were edited in SeqScape v.1.0 software, which was also used to obtain the consensus sequences. COI sequences were uploaded to the Barcode of Life Database platform (BOLD) which was used to estimate standard DNA barcode statistics such as the genetic distance analysis of Nearest Neighbor Distance (NND). The intra and inter genetic distances were calculated using the nucleotide evolution model Kimura 2-Parameter (K2P) [11]. The MEGA software v.6 [12] was used for distance calculations as well as the construction of dendrograms obtained by the NeighborJoining method (NJ) [13].

### Molecular data analysis

All individual sequences for each species were initially analyzed using the program Geneious Pro 5.4.2 [14] and consensus sequences were obtained. All sequences were aligned using MUSCLE [15], under default parameters and the alignment was inspected by eye for any obvious misalignments such as sequencing errors due to contamination, paralogy or pseudogenes. Nucleotide variation, substitution patterns, and genetic distances were examined using MEGA. The best-fit nucleotide evolution model for COI gene was evaluated under the information-theoretic measure of Akaike Information Criterion with corrections for small sample sizes (AICc). Genetic distance among specimens and species were calculated using MEGA 6.0 using the K2P model as the best model selected by MEGA 6.0. [12]. The dataset was combined with the following sequences available in the public reference database BOLD system (http://www.boldsystems.org): *Pogonias cromis* (code RFE, SMSA, GBMIN and GBGCA). A neighbour-joining (NJ), a maximum-likelihood (ML) and Maximum-Parsimony analyses were performed using MEGA. Robustness of trees was tested using bootstrap analysis with 1000 replicates. The nucleotide diagnostic (ND) approach was applied to strengthen the utility of the DNA barcoding technique to identify species. This approach is similar to the traditional morphology-based methods, where species identification would be based on the presence or absence of a distinct morphological feature. In ND analysis, a distinct DNA character will be assessed which is specific to the species, thereby preventing the ambiguity found in analogue measurement [16]. Automatic Barcode Gap Discovery (ABGD) algorithm was used the [17]; ABGD analysis was run on a matrix of pairwise distances, K2P nucleotide substitution model was selected. A default value was used (P min: 0.001-P max: 0.1; Steps = 10, Nb bins = 20 and X relative gap width = 1.5).

### Comparative material

*Pogonias cromis*: ANSP 162287, 1, 720 mm SL, Atlantic Ocean, United States of America. MLP 11338, 1, 198 mm SL, Apalachicola Bay, Gulf of Mexico, Florida, USA. MNHN A-8150, 1, 945 mm SL (dry mount), North America. MNHN A-880, 1, 153 mm SL, St. John´s river, USA. MNHN 0000–7575, 1, 177 mm SL, New York. MNHN 0000–7461, 1, 84 mm SL, Holotype *Pogonias fasciatus*, USA. MNHN 0000–7460, 1, 148.5 mm SL, New York, USA. UF 31219, 1, 700 mm SL, Atlantic Ocean, Florida District, USA. UNMDP 4878, 1, 254 mm SL, Palm River drainage, Hillsborough County, Florida, USA. UNMDP 4879, 1, 260 mm TL, St. Joseph Bay drainage, Gulf County, Florida, USA. USNM 142760, 1, 289 mm TL, Atlantic Ocean, Columbia District, USA. Fifteen specimens (500–980 mm TL) from Atlantic coast of República Oriental del Uruguay, obtained from commercial fisheries, examined in situ.

*Pogonias courbina*: **Argentina**, 54 specimens from provincia de Buenos Aires: MACN 4411, 1, 248 mm SL, Necochea, 38°33'S 58°44'W, E. Siccardi & Z. Popovici, no date. MACN 5085, 7, 170–195.5 mm (3 measured), Río Salado, 35°44'45''S 57°21'W, 1964, R. López. MACN 5981, 20 (no measured), 54.2–160.0 mm SL, San Clemente del Tuyú, 36°21'S 56°43'W, 1964, R. López. MACN 6295, 4, 73.3–85.0 mm SL, San Clemente del Tuyú, 36°21'S 56°43'W, 1964, R. López. MACN 11259, 2, 198.0–200.0 mm SL, provincia de Buenos Aires (Without other data or date). MLP 11337, 2, 79.0–97.8 mm SL, Bahía de Samborombón, 35°27'S 56°45'W, 1998, M. Azpelicueta. UNMDP 4848, 1, 307 mm SL, San Clemente del Tuyú, 36°21'S 56°43'W, 2017, M. Delpiani. UNMDP 4849, 1, 301 mm SL, San Clemente del Tuyú, 36°21'S 56°43'W, 2017, M. Delpiani. UNMDP 4850, 1 (measured), 488 mm SL, San Clemente del Tuyú, 36°21'S 56°43'W, 2017, M. Delpiani. UNMDP 4851, 1, 633 mm SL, Santa Teresita, 36°32'37''S 56°41'50''W, 2017, M. Delpiani. UNMDP 4852, 1, 660 mm SL, Santa Teresita, 36°32'37''S 56°41'50''W, 2017, M. Delpiani. UNMDP 4853, 1, 629 mm SL, Santa Teresita, 36°32'37''S 56°41'50''W, 2017, M. Delpiani. UNMDP 4872–73, 2, 301–307.8 mm SL, San Clemente del Tuyú, 36°21'S 56°43'W, 2018, M. Delpiani. UNMDP 4875–77, 3, 629–660 mm SL, San Clemente del Tuyú, 36°21'S 56°43'W, 2018, M. Delpiani. UNMDP 4880, 1, 855 mm SL, San Clemente del Tuyú, dry spines VII to XI (used in Fig 2). **Brazil**, 16 specimens: AZUSC 4271, 1, 447 mm SL, Estado de São Paulo, Ilha da Moeda, 24°3'4''S 46°15'36''W, 2014, M. A. Croce. AZUSC 4137, 3, 645 mm SL, Barra de Cananéia, 25°8'13''S 47°30'40''W, 2013, J. I. Medeiros. AZUSC 4232, 1, 525 mm SL, Barra de Paranaguá, 25°38'37''S 48°18'40''W, 2013, J. I. Medeiros. AZUSC 3772, 1, 370 mm SL, Baía de Santos, 24°0'14''S 46°21'3''W, 2012, M. A. Croce. AZUSC 4195, 1, 587 mm SL, Estado do Parana, Ilha da Figueira, 25°21'54''S 48°01'55''W, 2013, J. I. Medeiros. AZUSC 5037, 1, 204 mm SL, São Paulo, Peruíbe, Ilha do Ameixal, 24°15'48''S 46°12'W. AZUSC 4287, 1, 151 mm SL, São Paulo, Santos, Baía do Santos. LBP 21339, 1 (sequenced and x-rayed), 198 mm SL, Alto Estuário de Santos, 23°54'34''S 46°22'01''W, 2015, M. M. Rotundo. MACN 11766, 1, 117.5 mm SL, Brazil (without other data). MNHN A-950, 2, 172–182 mm SL. MNHN A-463, 1, 206 mm SL, Brazil. MNHN A-949, 2, 143–162 mm SL, Brazil. UNESP 120815, 1, 319 mm TL, São Paulo, 23°59'41"S 46°15'25"W**. Uruguay**, 2 specimens: MNHN 0000–9036, 1, 28.5 mm SL, Montevideo. UNMDP 4927, 1, 910 mm SL, Montevideo, Río de la Plata, 56°09'36"S 34°54'10"W, dry spines VII to XI (used in Fig 3), 1998, A. Cione.

#### Sample tissues used in this paper

Vouchers are deposited in the Laboratorio de Biotaxonomía Morfólogica y Molecular de Peces (BIMOPE), Universidad Nacional de Mar del Plata. DNA sequences were registered at GenBank and iBold. **Argentina**, provincia de Buenos Aires, Mar Chiquita coastal lagoon: INIDEP-T 0650, 1, 280 mm SL, 2008, M. González Castro. INIDEP-T 0651, 2, 281.0 mm SL, 2008, M. González Castro. INIDEP-T 0652, 3, 124.0 mm SL, 2008, M. González Castro. INIDEP-T 0287, 1, 342 mm SL, 2006, M. González Castro. INIDEP-T 0292, 2, 362 mm SL, 2006, M. González Castro. INIDEP-T 0298, 3, 385 mm SL, 2006, M. González Castro. INIDEP-T 0299, 5, 433 mm SL, 2006, M. González Castro. INIDEP-T 0300, 6, 359 mm SL, 2006, M. González Castro. INIDEP-T 0301, 7, 510 mm SL, 2006, M. González Castro. INIDEP-T 0302, 8, 474 mm SL, 2006, M. González Castro. INIDEP-T 0303, 9, 430 mm SL, 2006, M. González Castro. San Clemente del Tuyú: UNMDP 4848, 1, 307 mm SL, 2017, M. Delpiani. UNMDP 4849, 1, 301 mm SL, 2017, M. Delpiani. UNMDP 4850, 1, 488 mm SL, 2017, M. Delpiani. UNMDP 4872, 1, 301 mm SL, 2018, M. Delpiani. UNMDP 4873, 1, 307.8 mm SL, 2018, M. Delpiani. UNMDP 4875, 1, 633 mm SL, 2018, M. Delpiani. UNMDP 4876, 1, 660 mm SL, 2018, M. Delpiani. UNMDP 4877, 1, 629 mm SL, 2018, M. Delpiani. Santa Teresita: UNMDP 4851, 1, 633 mm SL, 2017, M. Delpiani. UNMDP 4852, 1, 660 mm SL, 2017, M. Delpiani. UNMDP 4853, 1, 629 mm SL, 2017, M. Delpiani. **Brazil**, São Paulo: LBPV-80865, 1, 198 mm SL, Santos, 2015, M. M. Rotundo. LBPV-91929, 1, 285 mm SL, Peruíbe, 2015, M. M. Rotundo.

## Results

*Pogonias courbina* (Lacepède, 1803)

*Pogonathus courbina* Lacepède 1803: 120. Type locality: Rio de la Plata [7].

*Pogonias chromis* var. *chromis*.-Berg, 1895: 57 [18].

*Pogonias chromis* var. *curbina*.-Berg, 1895: 58. Bahía Blanca, Mar del Plata,

Montevideo, Maldonado [18].

*Pogonias cromis*.-Marini, 1929: 454. Puerto Quequén (38°33′20″S 58°42′58″W) [19].

*Pogonias chromis*.-Pozzi & Bordalé, 1935: 168. Between 34° - 35° S, Atlantic Ocean [20].

*Pogonias chromis* var. *courbina*.-Pozzi & Bordalé, 1935: 168. Between 34° and 35° S, Atlantic Ocean [20].

*Sciena barbata* Larrañaga 1923: 380, 384. Uruguayan coast [21].

*Sciaena barbata*.-Devincenzi, 1925: 303. Coastal waters of Uruguay [22].

### Neotype

**Fig 1:**
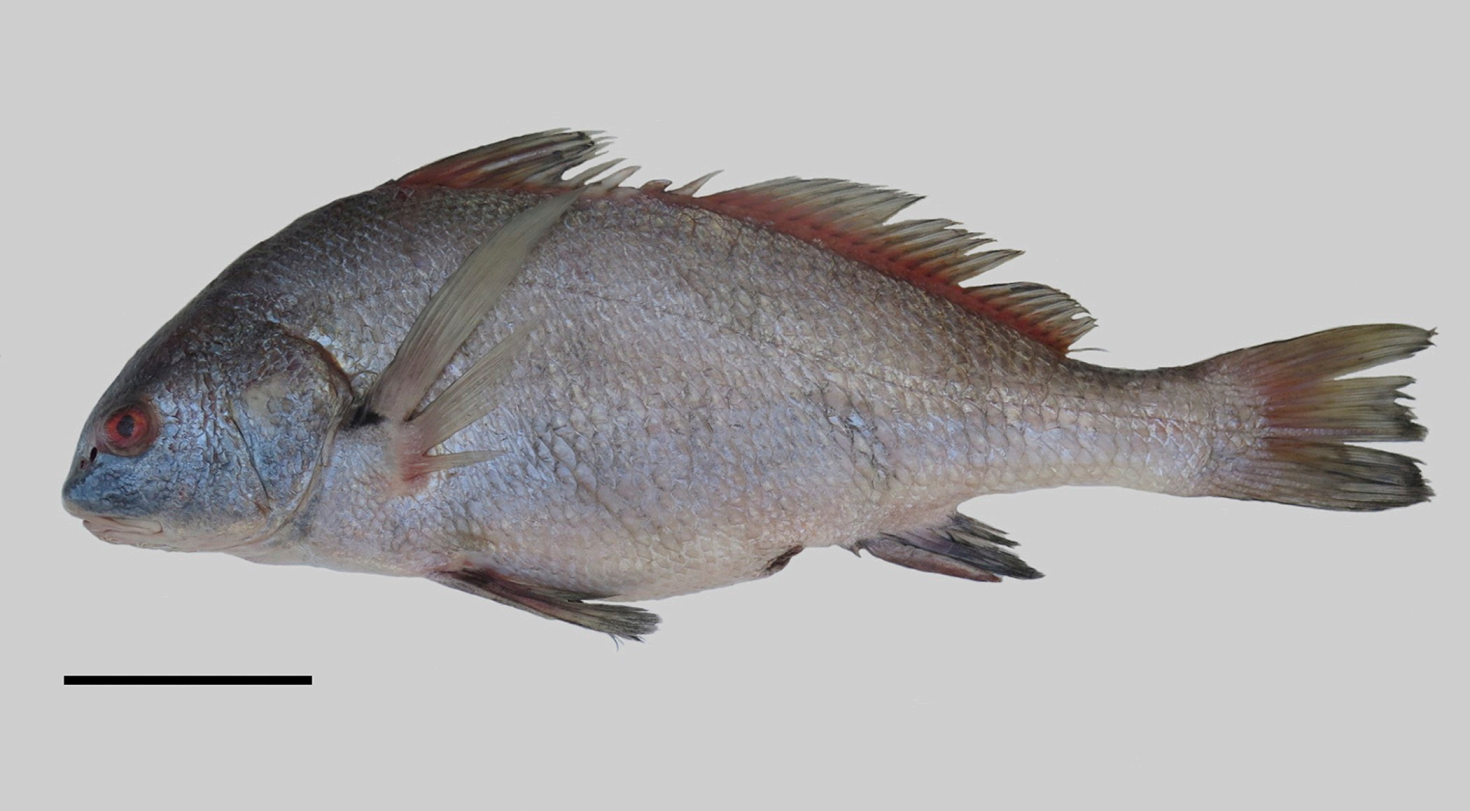
*Pogonias courbina*. **Neotype**. Argentina, Provincia de Buenos Aires, San Clemente del Tuyú, 488 mm SL. Scale bar = 10 cm.

UNMDP 4874, 488 mm, Argentina, Provincia de Buenos Aires, Atlantic Ocean in San Clemente del Tuyú (off Rio de la Plata), 36°21'S56°43'W. December 12, 2017, M. Delpiani (Fig 1).

### Diagnosis

*Pogonias courbina* is distinguished from *P*. *cromis* by the characteristic hyperostoses in the spines of the central part of dorsal fin of all specimens over 250 mm SL (vs hyperostose absent); by having all pterygiophores in the dorsal fin always laminar and thin (vs dorsal-fin pteygiophores broad); pterygiophores of anal fin slender excluded those of the anal spines (vs anal.fin pterygiophores broad); gas bladder with lateral processes simple (vs anterior and lateral processes complex); advertisement call short,120–200 milliseconds (vs advertisement call long, 600±22 ms). Genetic distance between *P*. *cromis* and *P*. *courbina* 1%. A compound nucleotide diagnostic discriminates between *P*. *cromis* and *P*. *courbina* by the exclusive occurrence of characters in six informative sites of COI (58 G; 214 G; 328 A; 331 A; 553C; 580 G).

### Description

Morphometric data for *P*. *courbina* and *P*. *cromis* in Table 1. Robust and large sized teleostean with maximum length of 1,170 mm [23]. Body oblong, moderately compressed, with very high profile. Dorsal profile of body strongly convex from snout tip to dorsal-fin origin, nearly straight under first dorsal-fin, slanting ventrally from this point to caudal peduncle, and nearly straight caudal peduncle. Ventral profile slanting ventrally to ventral-fin origin, generally straight or convex across abdomen to anal-fin origin, nearly convex over anal fin, and slanting dorsally to caudal peduncle with straight lower profile.

**Table 1:** Morphometric data of 30 specimens of *Pogonias courbina* and *P*. *cromis*.

	Argentina	N = 17			Brazil	N = 13	
	**Neotype**	**Range**	**Mean**	**SD**	**Range**	**Mean**	**SD**
Total length	590	97.8–860.0			11.0–627.0		
Standard length (mm)	488	79.0–660.0			94.2–516.0		
**Percentages of standard length**							
Head length	26.4	24.3–32.7	27.6	2.5	26.6–30.2	28.8	1.2
Snout length	6.9	4.8–7.5	6.1	0.8	4.5–7.5	6.39	0.7
Eye diameter	4.5	3.4–9.8	5.3	1.9	5.0–8.4	6.56	1.0
Posterior eye margin/dorsal-fin origin	20.3	15.8–23.2	19.2	1.9	14.2–21.5	18.5	2.0
Snout/dorsal-fin origin	36.1	32.1–43.1	38.2	2.4	33.0–42.0	38.6	4.5
Snout/pelvic-fin origin	34.0	29.8–36.6	33.0	1.6	31.0–36.0	33.8	1.3
Upper jaw length	9.7	8.6–12.2	10.2	1.2	9.6–10.4	9.8	0.2
Dorsal-fin origin/pelvic-fin origin	31.3	30.5–38.7	33.4	1.9	36.0–39.0	35.2	1.7
Pectoral-fin length	28.0	21.5–32.1	27.2	2.6	20.8–29.3	25.6	2.1
Maximum dorsal-fin 1 length	17.6	15.0–21.9	18.4	1.6	15.5–24.8	20.2	2.4
Dorsal-fin 1 base	25.1	22.5–25.8	24.4	1.1	30.3–35.8	33.8	2.4
Maximum dorsal-fin 2 length	16.3	11.1–20.2	14.7	2.0	11.0–18.2	15.2	1.4
Dorsal-fin 2 base	32.3	30.7–37.3	34.5	1.8	30.3–35.8	33.8	1.1
Caudal peduncle depth	9.2	9.1–12.5	10.2	0.8	9.0–13.0	10.3	1.5
Pelvic-fin length	18.7	18.7–24.8	21.0	1.9	17.6–24.0	21.7	1.1
Pelvic-fin origin/anal-fin origin	37.9	31.6–42.4	38.1	2.0	36.7–40.3	38.6	0.7
Anal-fin base	10.2	8.8–15.4	11.3	1.6	10.2–12.6	11.4	1.7
Anal-fin depth	18.3	16.2–22.2	19.0	1.5	18.0–22.8	20.2	1.0
**Percentages of head length**							
Eye	21.8	18.7–26.1	22.7	2.1	18.0–27.8	23.2	3.3
Snout	26.3	17.0–27.3	22.4	3.1	16.1–27.0	22.7	2.7
Interorbital	38.7	33.7–48.8	41.0	3.2	22.8–35.0	30.9	4.3

Maximum depth at dorsal-fin origin, approximately contained 2.5–3.0 times in SL. Maximum body width across head between opercles. Head relatively small and deep, contained 3.0–4.0 times in SL. Dorsum of head, opercle, preopercle, and cheek covered by ctenoid scales smaller than those of body. Depth of head at eye less than or equal to half maximum depth.

Snout short, blunt, its length scarcely shorter than eye diameter in small specimens and longer in large specimens; its length contained 3.6–5.6 times in HL, with relatively rounded profile in dorsal view. Anterior nostril rounded, posterior nostril ovate, smaller, and close to eye. Eye large in small specimens, smaller in larger specimens, placed laterally and anterior to midpoint of bony head length. Interorbital space strongly convex. Its width approximately 0.4–1.0 times in eye diameter. Mouth moderate in size, placed ventrally, lips slender. Upper jaw projecting, with mouth closed upper lips completely exposed.

Premaxillary teeth long, conical, slender, curved inward, arranged in three to five series; labial series longer than inner series. Median to large specimens with tip covered by enameloid detached by remarkable constriction. Dentary teeth conical, arranged in five to seven series; labial series slender and longer than those of outer series. No dentary or premaxillary fangs. No prevomerine or palatal teeth. Eleven to fourteen pairs of maxillary barbels, placed between symphysis and interopercle, in one cluster and two series. One series of six to eight pairs placed in soft tissue on dentary with posterior barbels longer than anterior ones. Other external series of three or four barbels developing around second pore of sensory canal. One anterior cluster of three to five small barbels sourrounding anterior opening of mandibular sensory canal, behind lip, near symphysis. All barbels with epidermical projections. Five pores of laterosensory mandibular canal. Branchiostegal membranes anteriorly joined to isthmus, diverging once without overlap. Six branchiostegal rays. Gill rakers on upper and lower limb of first arch, conical, with moderate size. Six to 8 gill rakers on upper limb, one at cartilaginous angle, and 16–17 on lower limb (ceratobranchial, cartilage, and hypobranchial, five specimens). Pharyngeal jaws remarkably robust with large grinding teeth set in broad series. Ceratobranchial 5 completely articulated in large specimens, not completely articulated in small and median specimens. Ceratobranchial 5 carrying several large polygonal teeth series at midline becoming smaller towards external margin series, where conical slender teeth developed. Number of tooth series increasing with age. Unworn crushing teeth with enameloid central peak. Upper pharygobranchials 2 to 4 with teeth, especially large in pharyngobranchial 3. Teeth with similar shape and pattern than those of ceratobranchial 5.

**Fig 2:**
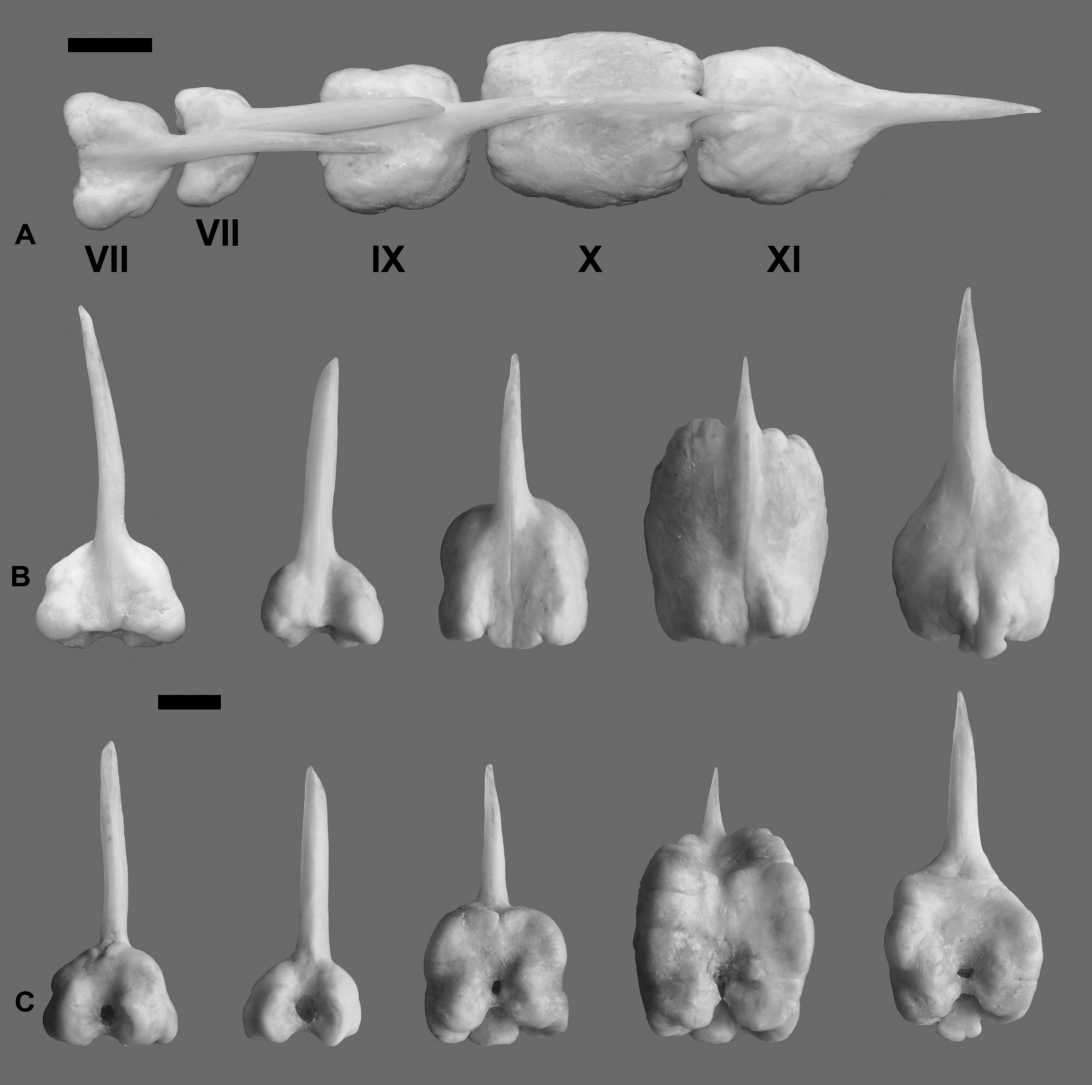
*Pogonias courbina*. **Enlarged spines VII-XI of dorsal fin. UNMDP 4880, 855 mm SL**. (A) Position in the specimen, dorsal view. (B). Detail of each spine in dorsal view. (C). Detail of each spine in ventral view. Scale bar = 1 cm.

**Fig 3:**
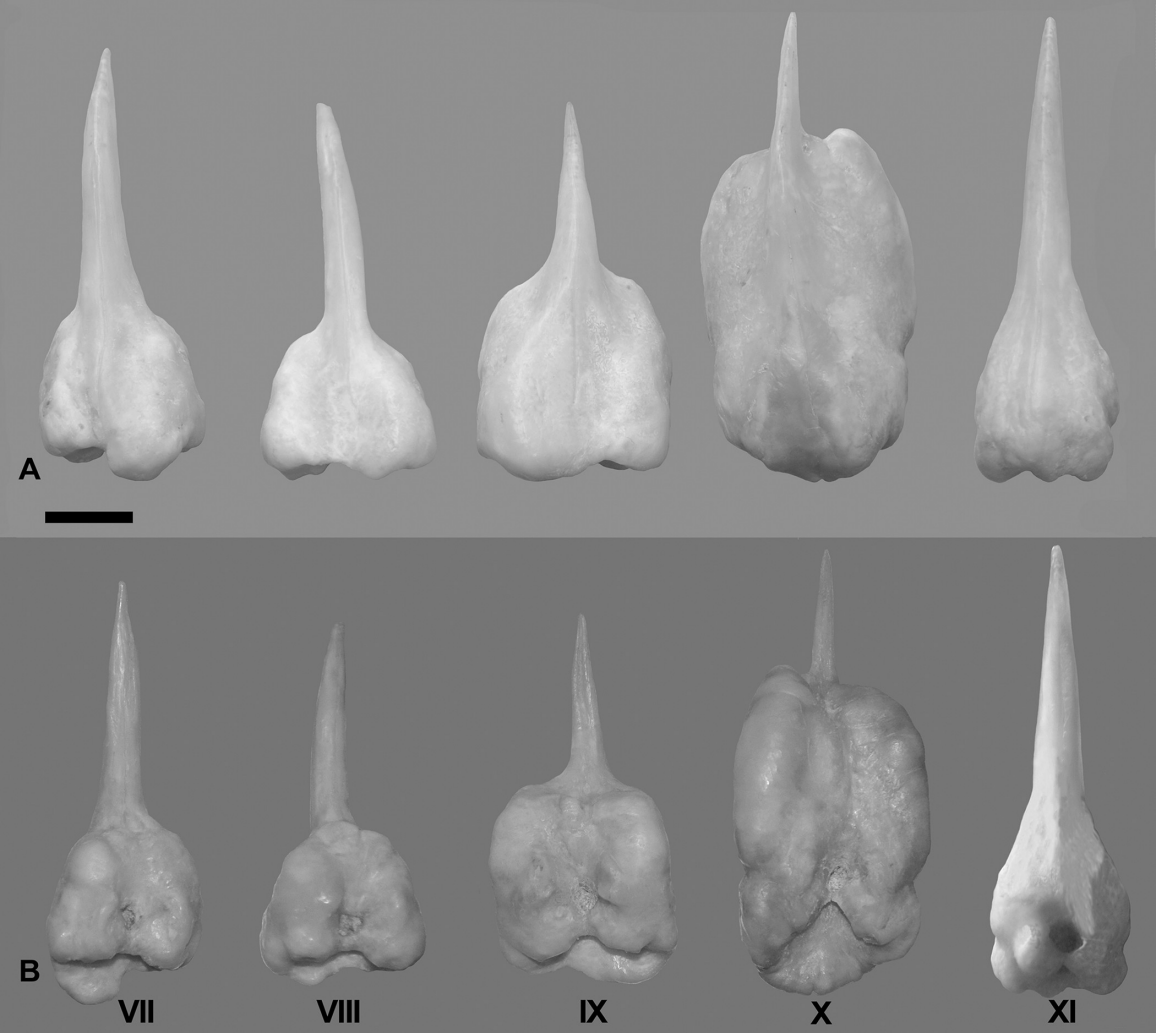
*Pogonias courbina*. **Enlarged spines VII-XI of dorsal fin. UNMDP 4927, 910 mm SL**. (A). Detail of each spine in dorsal view. (B). Detail of each spine in ventral view. Scale bar = 1 cm.

**Fig 4:**
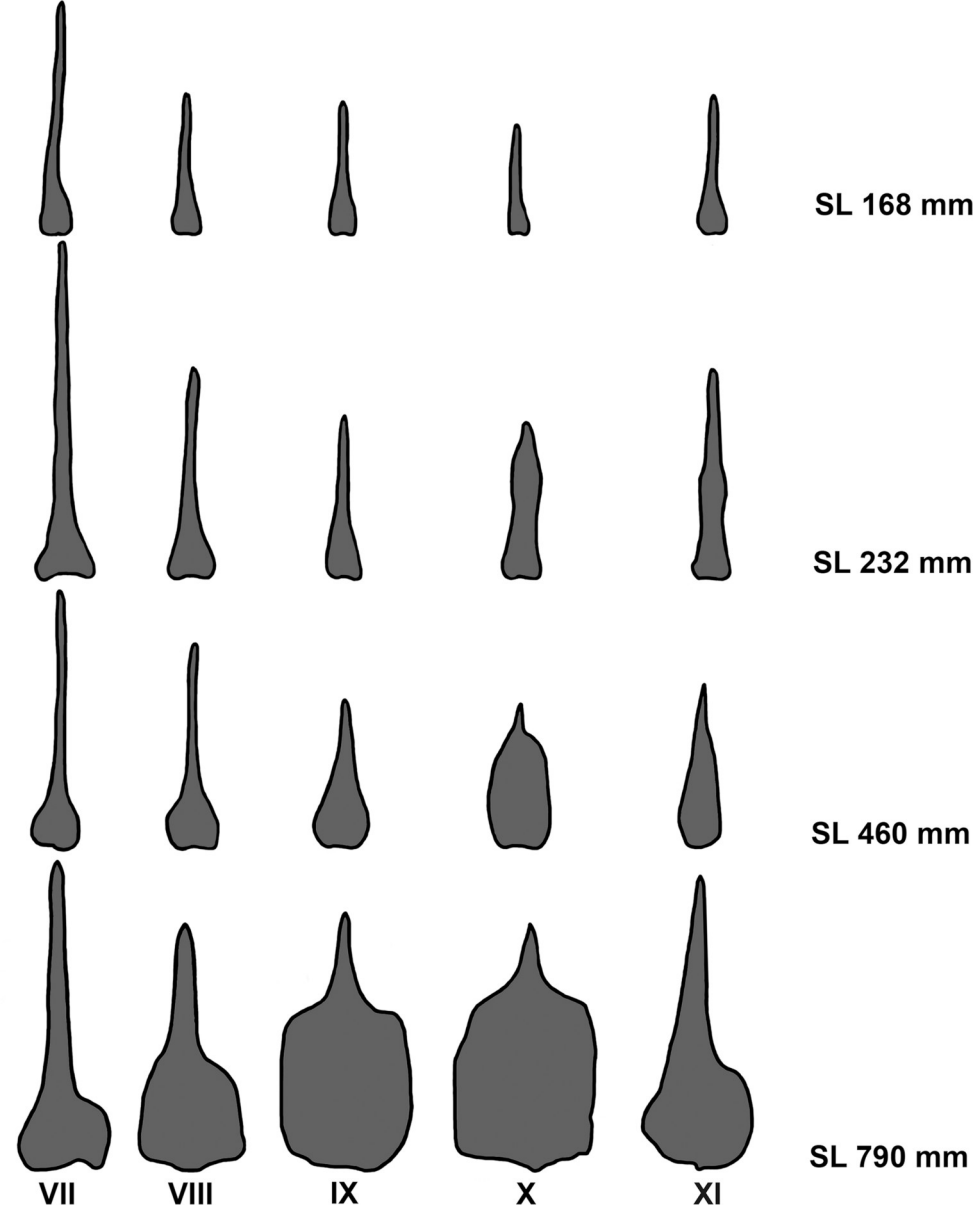
Ontogenetic variation in shape of spines VII-X of dorsal fin 1 and spine XI of dorsal fin 2, schematic drawings. Length of different size specimens at right.

**Fig 5:**
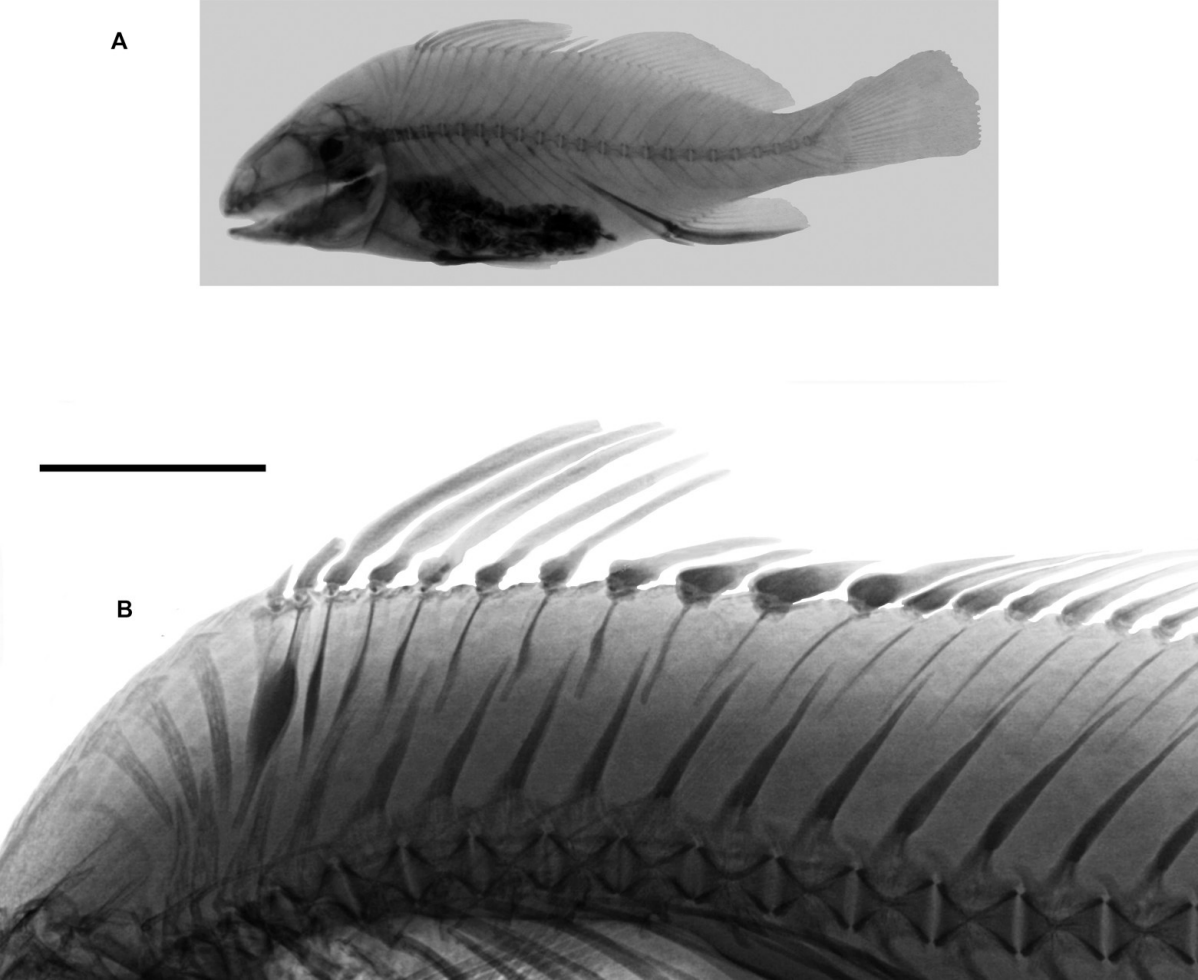
*Pogonias courbina*. (A). Radiograph of a juvenile (103 mm SL). Note the absence of ray enlargements. (B). Radiograph of an adult (471 mm SL). SP VII-XI: spines VII to X of dorsal fin 1 and spine XI of dorsal fin 2. Specimens not preserved. Scale bar = 5 cm.

Deep notch separating anterior and posterior dorsal fins. First dorsal fin with X spines; second dorsal fin commencing with spine number XI always and bearing 19*-23 branched rays. First spine small, short, triangular in shape. Second spine longitudinally striated especially in medium-sized specimens, lying on largest segment of third basal. Spines III and IV longest; spines I to VI with similar shape during ontogeny, slender as common spiniform ray. Spines VII and VIII but especially IX, X, and XI with remarkably differential growth during ontogeny in both sexes (Figs 2, 3, 4 and 5). Expansions frequently asymmetrical in all spines. Expansion of basal portion of spine making it convex instead of concave, in some large specimens. Spines VII and VIII moderately expanded, only at their bases. Spine IX vaguely quadrangular in adults with spiniform projection (actually unmodified distal part of ray), approximately equal in length to expanded area; its strong expansion in mid-diameter, modifying completely shape of spine. Base of spines bearing articular area, formed by two rostral condyles. Anteriorly, spine with slightly deep, longitudinal groove; distal portion of spine VIII lying in this groove (Figs 2 and 3). Distal portion of spine IX bending ventrally. Adult spine X largest, usually somewhat rectangular body, with anterior longitudinal crest continued in distal short and unexpanded spiniform portion. Basal portion with anterior shallow groove, receiving distal part of spine IX (Fig 2). First spine of second dorsal fin expanded at base, with variably elongated spiny dorsal portion (unexpanded area, Figs 2 and 3). Shallow anterior longitudinal groove in anterior basal portion. Dorsal pterygiophores laminar (Fig 5).

Dorsal-fin origin scarcely displaced posteriorly to vertical through pectoral-fin origin, more distant in larger specimens. First dorsal fin triangular in shape, second dorsal fin almost rectangular, with upper margin scarcely convex.

**Fig 6:**
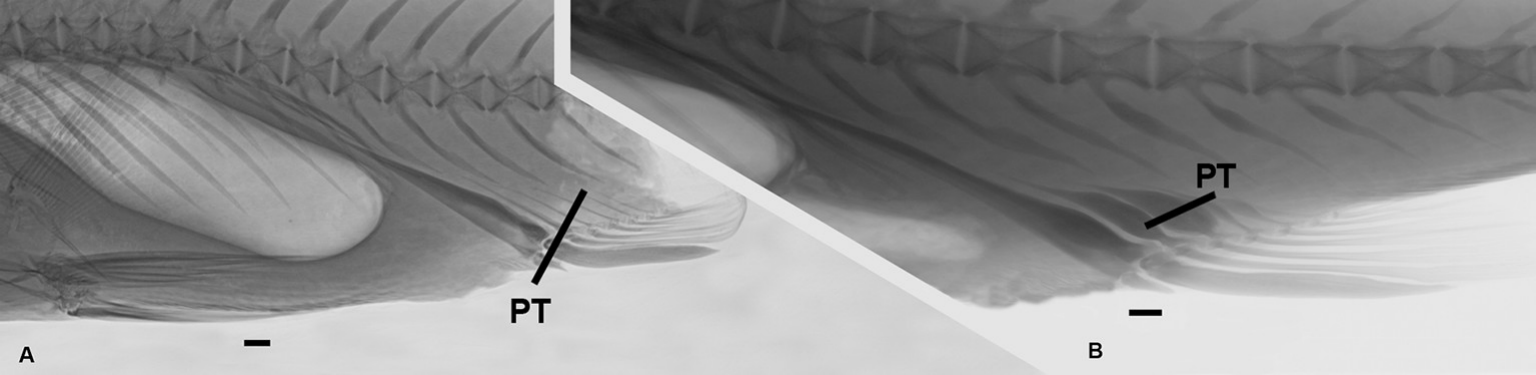
Anal-fin pterygiophores. (A). *Pogonias courbina*, 680 mm SL. (B). *Pogonias cromis*, 720 mm SL. PT: pterygiophores. Scale bar = 1 cm.

Distal margin of pectoral fin almost straight. Pectoral fin with i,15–19* rays. First four or five rays longer than remaining rays and sometimes filamentous. Pectoral-fin origin very close to opercle, five scales below lateral line. Pelvic-fin origin scarcely posterior to vertical through dorsal-fin origin, rhomboidal in shape, tip falling four or five scales before anus; pelvic fin with I,5* rays. Anal-fin origin located at vertical between rays 17 and 20 of second dorsal fin; tip of anal fin reaching middle caudal peduncle; anal fin with II,4-7(6*) rays. First spine very short, second spine striated, very large, and robust. Anal-fin pterygiophores laminar (Figs 5 and 6) excluded those of spines. Caudal-fin margin almost straight; with 1,15–18,1 rays.

Lateral line complete, almost parallel to dorsal-fin base and ending near end of middle caudal-fin rays, 51*-56 perforated scales. Line of about 20 very small perforated scales developed after origin of middle caudal-fin rays. Transverse scales: 5–8* dorsal scales, 8–11 ventral scales, 10*. Total number of vertebrae, 23–24 (n = 3 radiographs); 10–11 precaudal and 13 caudal; 9 pairs of ribs (n = 3).

**Fig 7:**
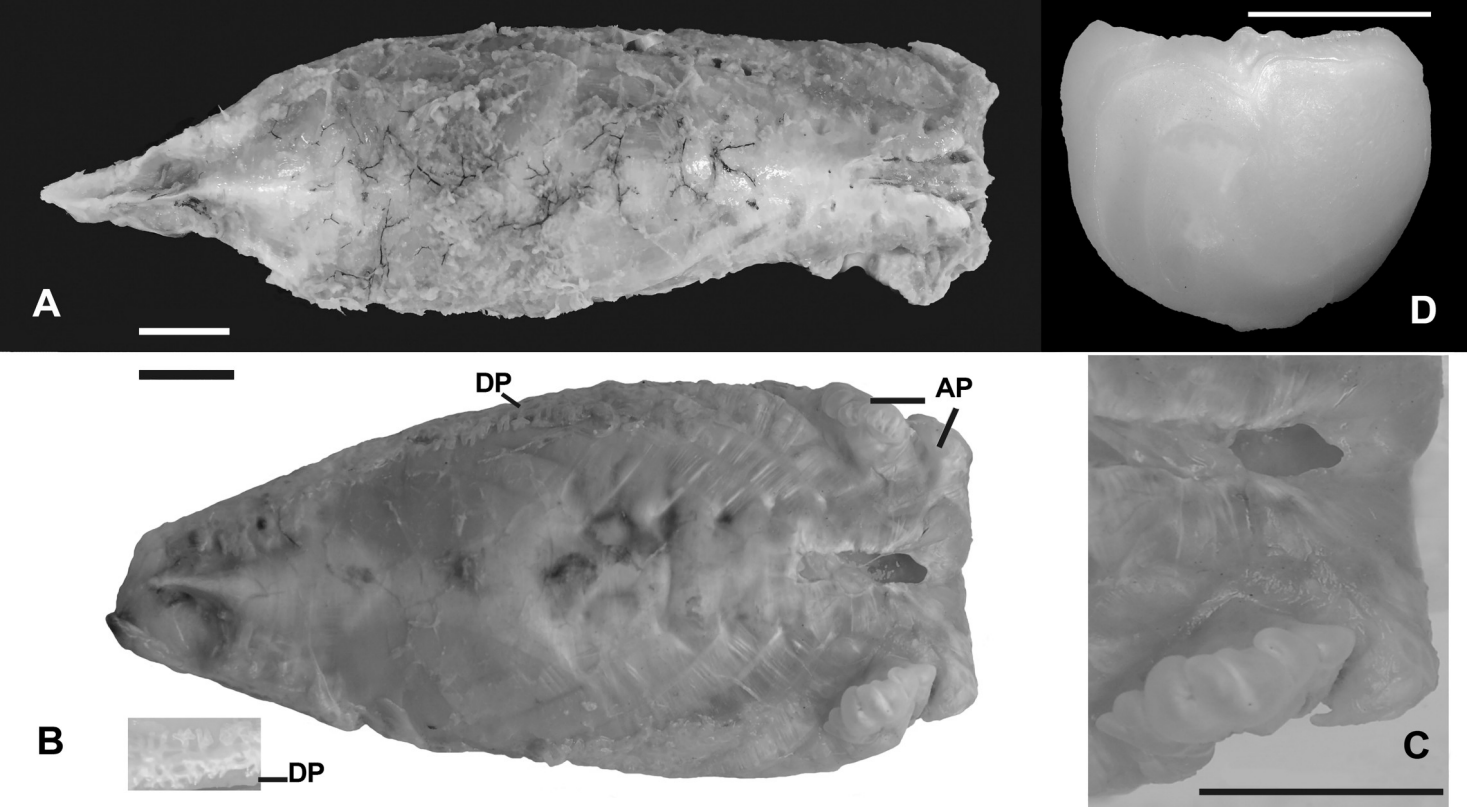
Gas bladder of *Pogonias courbina* and *P*. *cromis*. (A). *Pogonias courbina*, UNESP 1120815, 319.0 mm TL. (B). *Pogonias cromis*, UNMDP 4879, 300 mm TL. Scale bar = 1 cm. (C). Detail of the antero-dorsal processes in *P*. *cromis*. AP: Antero-dorsal process; DP: Dendriform projections; (D) Sagitta of *P*. *courbina*. Scale bar = 1 cm.

Gas bladder ellipsoid in shape with anterior portion straight and relatively far from ear; posterior tip acute, reaching anal-fin pterygiophores. One triangular, thin, laminar process at each side, in anterior half of gas bladder in small specimens at (130 mm SL). Larger specimens with lateral process increasing its size reaching distal end, forming also few finger-like projections laterally developed (Fig 7). Ventral surface smooth. Males and females with sonic muscles.

Sagitta somewhat rectangular, with dorsal margin slightly concave; most specimens with small process at middle portion of this margin. All remaining margins with variable undulations, more notable in smaller specimens, and especially in anterior margin. Ostium large, occupying anterior half of otholit almost completely; sulcus short; cauda and sulcus forming angle of 90°; tip of cauda always developed close to ventral margin (Fig 7D).

### Coloration

**Fig 8:**
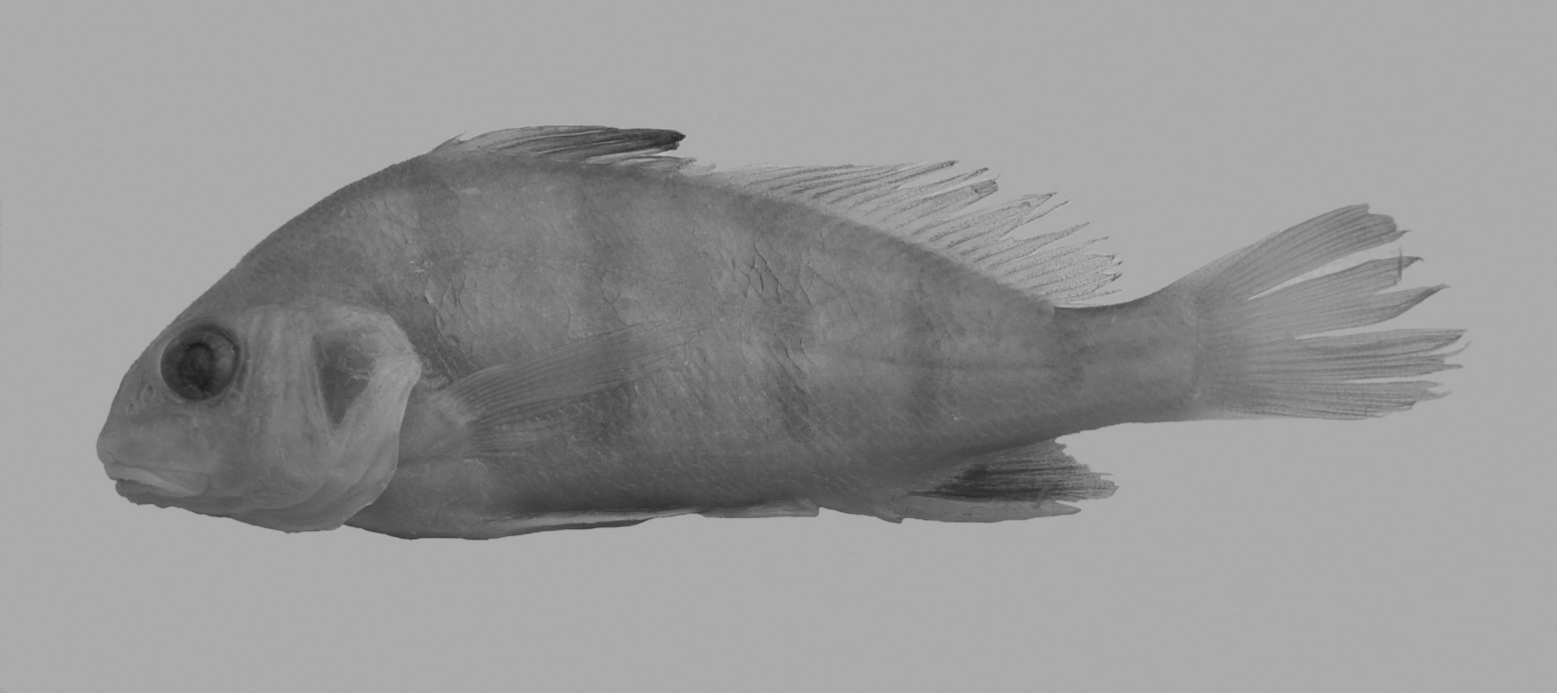
*Pogonias courbina*. Small specimen with black bars on flanks, 120.2 mm SL.

Ground color of body silver; flanks with four or five black bars, especially remarkable in young specimens (Fig 8). Those bars usually diffuse or absent in many juvenile specimens of different lengths and also in large specimens (Fig 2). First bar located at origin of first dorsal fin; last bar located around caudal peduncle. Dorsal part of head and dorsum mostly black or dark gray. Fins light to dark gray in color. Some specimens with base of dorsal and caudal fins light red, and less intense red in pectoral, pelvic, and anal fins. Differences in coloration not evident in individuals collected in separate areas sampled.

### Molecular data

**Fig 9:**
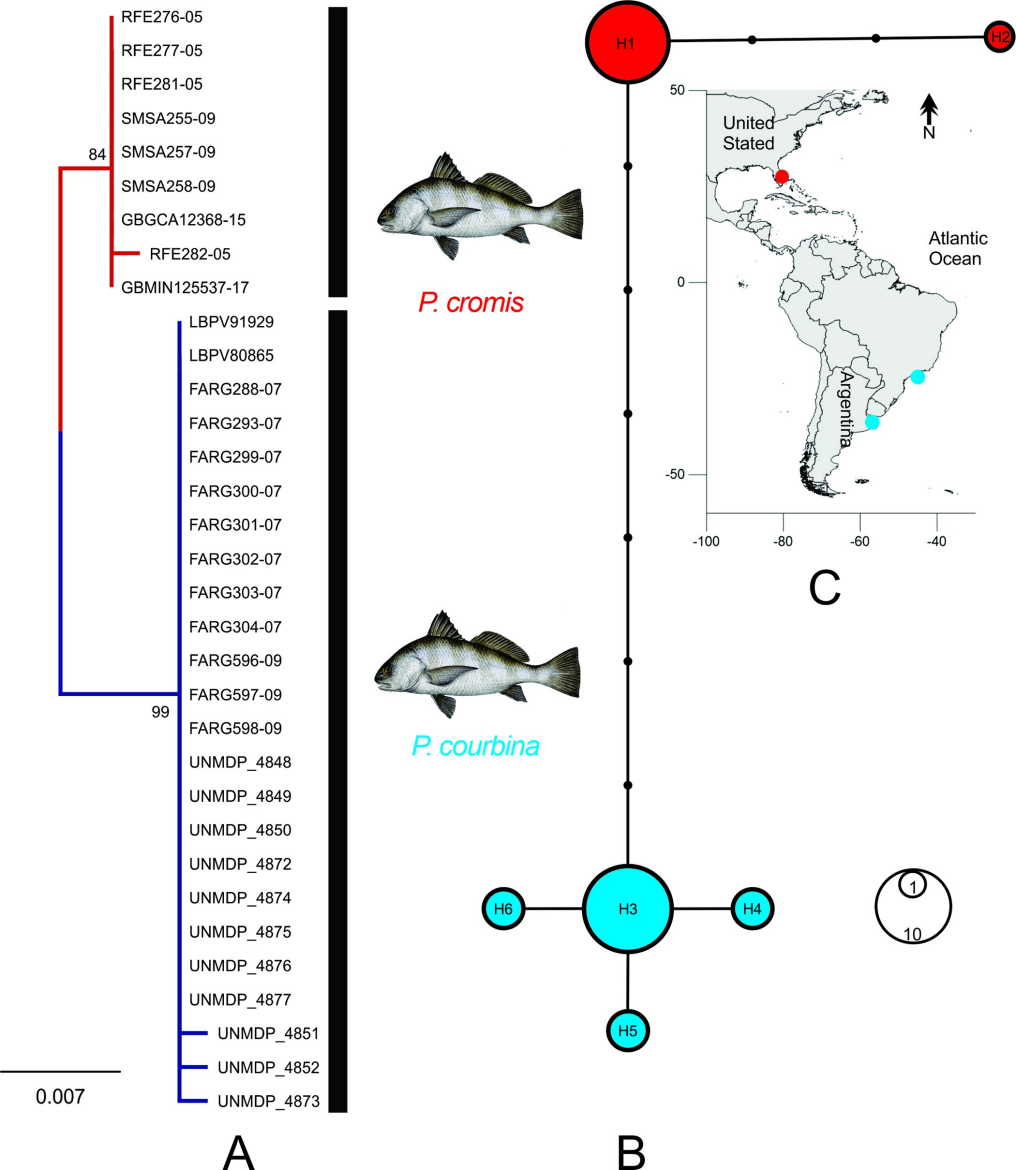
Phylogram built from the mitochondrial COI sequences of *Pogonias courbina* and *P*. *cromis*. (A). Statistical node support is shown as ML bootstrap. Sequences of *Sciaenops ocellatus* were used as outgroup (not shown) and vertical blackbars review the partition obtained according to Automatic Barcode Gap Discovery (ABGD). (B). Median-joining network of *P*. *cromis* and *P*. *courbina* haplotypes, size is proportional to their frequencies. (C). Geographical location of sampled specimens of *Pogonias*. The map was performed using the R statistical software, version 3.1.0.

Sequence data for 603 bp fragment of COI were obtained for 34 specimens. The best-fit model of evolution for the data was K2 model with AICc = 1899. All sequences showed no insertions, deletions, stop-codons or sequencing errors due to contamination or paralogy. The nucleotide frequencies were A = 24.52%, T/U = 27.60%, C = 29.52%, and G = 18.36%. The NJ, ML (based on K2P model) and MP analyses generated trees with nearly identical topologies. Interestingly, no shared haplotypes were found between *P*. *cromis* and *P*. *courbina* specimens. Moreover, a compound nucleotide diagnostic discriminates between *P*. *cromis* and *P*. *courbina* by the exclusive occurrence of characters in six informative sites (58 G; 214 G; 328 A; 331 A; 553C; 580 G). Sequence analysis revealed nine variable sites corresponding to four haplotypes, corresponding two haplotypes for each species (Fig 9). Considering sequences of two species from the Atlantic, the genetic distances between species was 1 ± 0.4%. ABGD method formed 2 groups with a maximum intragroup divergence P = 0.0077, discriminating two putative species of *Pogonias*. The ABGD partitioning was consistent with the topology of the ML tree (Fig 9A). Furthermore, no shared haplotypes between *P*. *cromis* and *P*. *courbina* were found (Fig 9B), and clearly correlated with the geographical distribution (Fig 9C). Besides compound nucleotide diagnostic character analysis supports the discrimination between *P*. *cromis* and *P*. *courbina*.

### Distribution

*Pogonias courbina* is a demersal coastal species distributed along the Southwestern Atlantic Ocean from the State of Rio de Janeiro (Brazil) to the south of Golfo San Matías, in Argentina. It is an estuarine-dependent marine species [23, 24] and is the largest sciaenid observed in estuarine nursery areas such as Río de la Plata and Mar Chiquita coastal lagoon (close to Mar del Plata city) where it is one of the most abundant fish.

## Discussion

Only two species of the black drum genus *Pogonias* were described from Southwesten Atlantic populations, *Pogonias courbina* Lacepède 1803 (as *Pogonathus courbina*) and *P*. *barbata* Larrañaga 1923 (as *Sciaena barbata*) [7, 21], both descriptions based on material from the Rio de la Plata. The genus *Pogonathus* has been later synonymized as *Pogonias* [25] and both species were assigned as synonyms of *P*. *cromis*.

Following the Lacepede's text provided in Histoire Naturelle des poissons, the specimen of *P*. *courbina* was collected by Commerson in 1767, in the Río de la Plata and it was never illustrated. The specimen unequivocally belongs to the genus *Pogonias* by the large number of mandibular barbels; Lacepède mentioned the presence of 24 barbels. *Pogonias* is the only genus with that number of barbels in Southern South Atlantic. In addition, Lacepède provided other diagnostic features that strongly support that his described specimen is a *Pogonias* species. Comparisons of meristics between the described specimen of Lacepède and specimens studied in this paper reveal many similarities. Those features of *Pogonias courbina* (24 barbels at lower jaw, 18 pectoral-fin rays, one spine and 5 pelvic-branched rays, 22 rays on second dorsal-fin) are within ranges for specimens of *Pogonias* reported here (22–28 barbels, 16–18 pectoral-fin rays, i5 pelvic-fin rays, 19–23 second-dorsal fin rays). In his *Pogonias* group diagnosis Chao [25] stated lower jaw with five pores and 12 to 13 pairs of small barbels. Based on morphological and molecular features, the specimens of *Pogonias* from Southern Atlantic Ocean are in fact a different species of *P*. *cromis*. Subsequently, *P*. *courbina* is the oldest available name for identifying the new species. The absence of type material made possible the designation of a neotype, following the recommendations of the ICZN (art. 75) [26]. Although a precise locality for *P*. *courbina* is unknown, the neotype was certainly collected close to the original locality given the distribution of the species in the Rio de la Plata estuary (art. 76) [26]. Larrañaga [21] also stated a high number of mandibular barbels.

**Fig 10:**
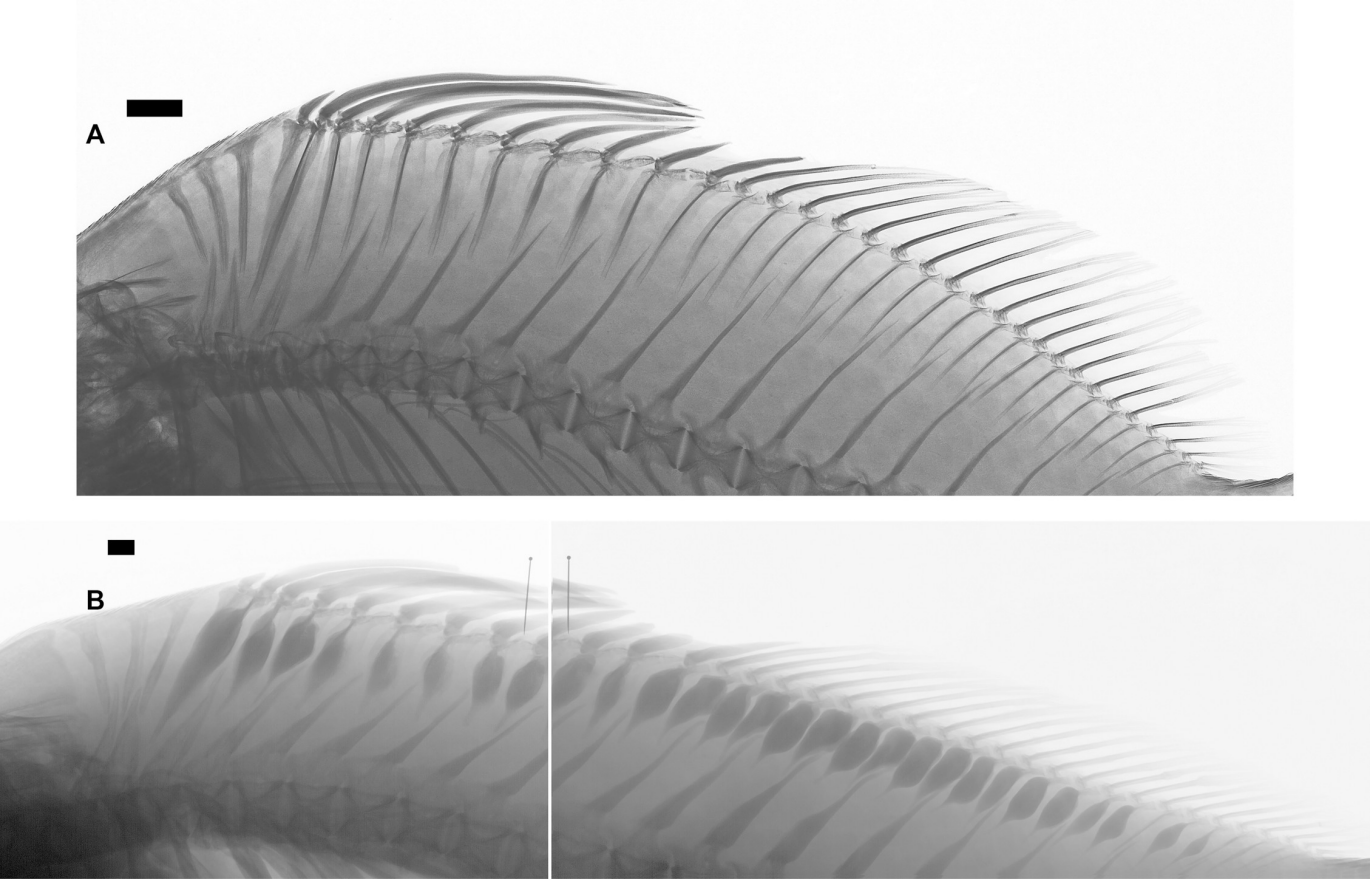
*Pogonias cromis*. (A). USNM 142760, radiograph of an adult, about 300 mm SL. Note the absence of ray enlargements. (B). ANSP 162287, Radiograph of an adult, 720 mm SL. Note modifications in the size of dorsal-fin pterygiophores. Scale bars = 1 cm.

*Pogonias courbina* differs from its congener *P*. *cromis* by the occurrence of characteristic hyperostoses in the spines of the central part of dorsal fin of all specimens over 250 mm SL, with several changes during growth (Figs 2, 3, 4 and 5), whereas they are absent in *P*. *cromis* (Fig 10). In addition, the dorsal-fin pterygiophores in *P*. *courbina* are laminar and thin whereas those of *P*. *cromis* are broad (Fig 10B); the pterygiophores of anal soft rays are slender in *P*. *courbina* and those anal-fin pterygiophores are robust in large *P*. *cromis* (Fig 6).

The morphology of the gas bladder differs in both species. *Pogonias courbina* has lateral projections with few finger-like structures, increasing in length according to growth; antero-dorsal projections were never found. *Pogonias cromis* has lateral projections with very numerous dendriform structures forming a complex pattern, increasing in size and complexity during growth (Fig 7B and 7C; figure 10 of Chao [25]; figure 68 of Sazaki [27]). Also, two antero-dorsal rounded projections are present in *P*. *cromis*, and the posterior one is larger and flower-shaped (Fig 7B and 7C).

In addition, both species differ in the duration of advertisement calls, three times shorter in *P*. *courbina* (call durations between 120 and 200 pulses measured in ms) in comparison with calls three-fold longer in *P*. *cromis* (600±22 ms) [28].

**Fig 11:**
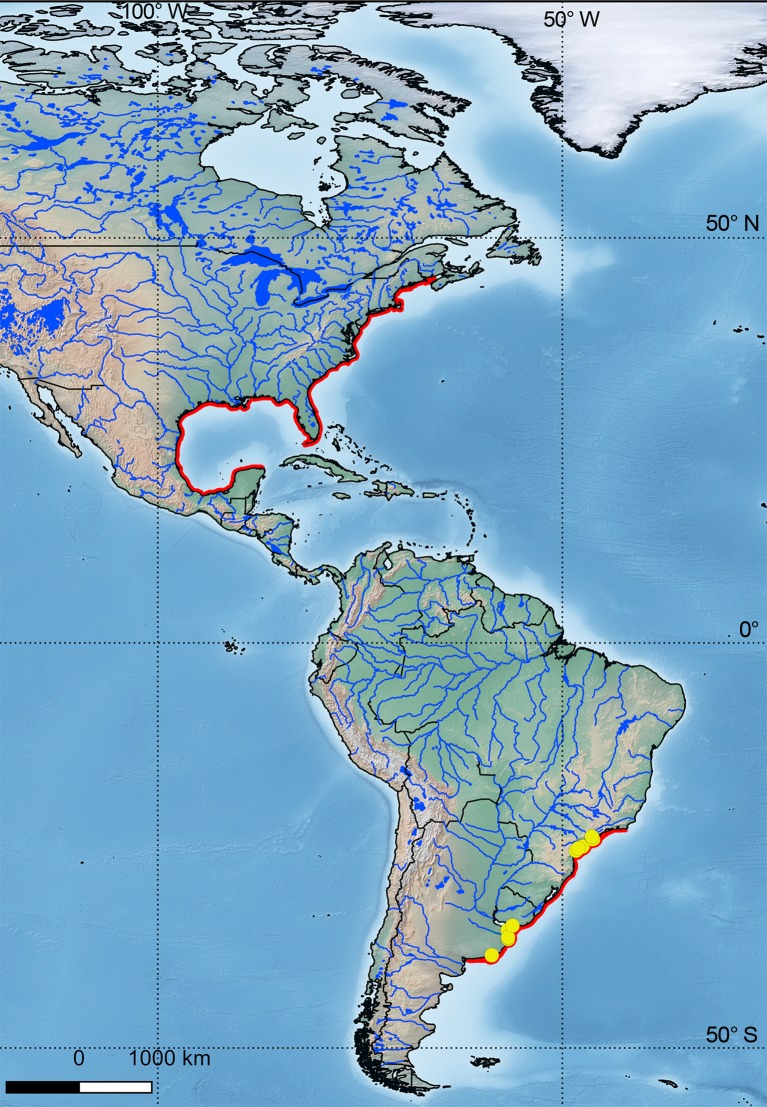
Geographical distribution of *Pogonias courbina* and *P*. *cromis* showing the gap between both species. The map was made using Natural Earth Data and QGIS 2.18.13. *Pogonias courbina* can reach a maximum size of 1170 mm (TL); the species live as long as 45–55 years and mature around 4–5 years of age in the la Plata River estuary [23]. Maximum weight known is 48.1 kg; maximum age is 57 years [23] and maturity is reached at 3–4 years in southern Brazil (Haimovici, unpublished data fide [31]). In North America, the maximum size of *P*. *cromis* is approximately 1700 mm [32] and its weight as much as 51.3 kg [33]. The maximum age estimated is 58 years [34].

Besides, distribution of both species appears to be separated by a gap of about 8000 km along the Western Atlantic Ocean. Only two reports were found in the gap: larvae attributed to *Pogonias* in tropical Brazil (Fig 11) [29, 30].

### Molecular analysis

The molecular results showed that *P*. *courbina* truly represents an individual species different of *P*. *cromis* (Fig 9). In specific groups, such as the Neotropical fishes, studies have used the barcoding threshold around 2% [35, 36, 37]. Nevertheless, given the great diversity of species, particularly related to life histories and environment complexity, a fixed threshold value might under- or overestimate the actual number of species [38] and the genetic distance of 1% between *P*. *cromis* and *P*. *courbina* suggests that the two species are taxonomically distinct. *Pogonias cromis* and *P*. *courbina* were discriminated into two putative species in ABGD tool which was also confirmed by pair wise K2P distance estimation and NJ, MP and ML tree analysis. Number of DNA sequences in Table 2.

**Table 2:** Numbers of DNA sequences deposited in GenBank; numbers with an asterisk corresponds to ID of iBold.

Species	Specimen code	Number
*Pogonias cromis*	GBGCA12368-15	KP722765
*Pogonias cromis*	GBMIN125537-17	KX164000
*Pogonias cromis*	RFE276-05	EU752167
*Pogonias cromis*	RFE277-05	EU752164
*Pogonias cromis*	RFE281-05	EU752165
*Pogonias cromis*	SMSA255-09	JQ842656.1
*Pogonias cromis*	SMSA257-09	JQ842654.1
*Pogonias cromis*	SMSA258-09	JQ842655.1
*Pogonias cromis*	RFE282-05	EU752166
*Pogonias courbina*	LBPV80865	MK834248
*Pogonias courbina*	LBPV91929	MK834249
*Pogonias courbina*	FARG288-07	EU074550
*Pogonias courbina*	FARG293-07	EU074549
*Pogonias courbina*	FARG299-07	EU074548
*Pogonias courbina*	FARG300-07	EU074547
*Pogonias courbina*	FARG301-07	EU074546
*Pogonias courbina*	FARG302-07	EU074545
*Pogonias courbina*	FARG303-07	EU074544
*Pogonias courbina*	FARG304-07	EU074543
*Pogonias courbina*	FARG596-09	*FARG596-09
*Pogonias courbina*	FARG597-09	*FARG597-09
*Pogonias courbina*	FARG598-09	*FARG598-09
*Pogonias courbina*	UNMDP_4848	MK834237
*Pogonias courbina*	UNMDP_4849	MK834238
*Pogonias courbina*	UNMDP_4850	MK834239
*Pogonias courbina*	UNMDP_4872	MK834240
*Pogonias courbina*	UNMDP_4874	MK834241
*Pogonias courbina*	UNMDP_4875	MK834242
*Pogonias courbina*	UNMDP_4876	MK834243
*Pogonias courbina*	UNMDP_4877	MK834244
*Pogonias courbina*	UNMDP_4851	MK834245
*Pogonias courbina*	UNMDP_4852	MK834246
*Pogonias courbina*	UNMDP_4873	MK834247

### Sound production

Sciaenids produce sound by contraction of specialized extrinsic sonic muscles attached to the swim bladder, thus they are called croakers or drums [28]. Males and females produce disturbance sounds when handled. During the reproductive season, only males of black drums produce sounds as advertisement calls used in courtship [34, 39]. These generated sounds may have high amplitude, exceeding 160dB re: 1 μPa [28, 40]. Tellechea et al [28] studied the sound production patterns of *P*. *courbina* from the Río de la Plata in Uruguay. Each call consists of short duration pulses, with an average of 23ms between pulses. During growth, the duration of pulses increases and the frequency decreases. The advertisement calls of the black drum *P*. *courbina* in Uruguay have shorter duration of calls, of about 120 to 200ms, while populations of *P*. *cromis* from Florida have longer call durations, of about 600 to 722 ms [28, 41, 42]. Tellechea et al suggested that this difference found in the duration of advertisement calls indicates genetic differentiation between northern and southern individuals [28].

### Geographical distribution

Different authors [31, 43] indicated the likely existence of two populations of *Pogonias*, considering an anti-tropical distribution which parallelize with the geographical distribution of the sciaenid genus *Micropogonias* (the southern species *M*. *furnieri* and the northern species *M*. *undulatus*). Haimovici and Klippel [44] include *P*. *cromis* in fishes of the southeast and south regions of Brazil, not to the north. The gap between the North and South Atlantic species of *Pogonias* involved coastal areas and estuaries of Central America, Venezuela [45], Antilles [46], French Guaiana [47], Surinam [48], Amapá state [49], Pará state [49, 50], mouth of Amazon River, Pará state [50]; Rio Grande do Norte state [51]. Marceniuk et al [52] confirms the gap in the North of Brazil. The gap would be as large as 8000 km (Fig 11).

However, some rare reports in the gap include three larvae in the “Santa Cruz Channel,” a tropical mangrove estuary in State of Pernambuco [29] and a 30 mm specimen attributed to *P*. *cromis* (0,03% of the total sample), cited for the Mamanguape River estuary in State of Paraiba [30].

In the southwestern Atlantic *P*. *courbina* specimens are relatively common from São Paulo state in Brazil to northern Río Negro province (San Matías Gulf, 42°S) in Argentina [53, 54]. It is a typical species of the Argentinian Biogeographic Province sensu López [55]. Like many other sciaenids, juveniles of *P*. *courbina* live in estuarine areas (e.g., Lagoa dos Patos (Brazil), Laguna de Rocha (Uruguay), Río de la Plata and Mar Chiquita coastal lagoon (Argentina) where they can tolerate a wide range of salinities and water temperatures [56]. Juveniles move into marine waters when fish reach the adult stage [57].

### Bathymetric distribution

Nion et al. [58] studied the bathymetry of black drums in the Río de la Plata area, reported a range of 4.2 to 14 m. However, Norbis et al. [59] reported specimens ranging to 30 m. Juveniles occurred in tides pools in rocky coasts of the Río de la Plata [60]. It is also a common and abundant species in Mar Chiquita coastal lagoon -a World Reserve of Biosphere- where it occurs at depths less than 2 m [23].

### Abundance

The black drum *P*. *courbina* is the most endangered sciaenid in the South Western Atlantic. In Brazil, captures were more abundant in the State of Santa Catarina than in the northern State of São Paulo. In Santa Catarina the resource is overfished; the reduction in tons is remarkable from 1976 (1450 tons) to 1990 (81 tons). In Uruguay, landings were variable through years, 120 t in 1977 to over 692 t in 2003 [31]. In Argentina, artisanal and sport fisheries produced 12 to 271 t between 1991 and 2007, and 136 t in 2017. This species was not assessed as threatened in Argentina [61]. Nonetheless, in the IUCN Red List, it is considered Endagered in the southern Atlantic Ocean (Brazil, Uruguay, Argentina) with specimens from Brazil as Critically Endangered [62].

In Uruguay and Argentina, the most important captures take place between December and January during the spawning season in the mouth of the Río de la Plata. *Pogonias courbina* is commercially harvested in inshore waters of Samborombón Bay, a semi-enclosed region inside the Río de la Plata estuary. Because of the importance of the artisanal, recreational, and commercial fisheries that exploit this resource, it is extremely necessary to improve management for fisheries of *Pogonias* in coastal areas of Southern South America. Regulations are needed especially to stop overexploitation during spawning and breeding seasons.

### Reproductive biology

The black drum *P*. *courbina* of the Río de la Plata estuary is a multiple spawner. In this region black drums aggregations have been observed at Samborombón Bay in depths of less than 10 m, mainly from October to December (Spring season). This period was considered the main spawning period for black drum aggregations in the Samborombón Bay, based on macroscopic and histological analysis of gonads [4].

*Pogonias cromis* is group-synchronous, broadcast spawners, aggregating in the spring near mouths of bays and rivers. *Pogonias cromis* spawns from April to June, in nearshore waters, especially in bays and estuaries [63, 64]. Spawning in the mouth of the Chesapeake Bay [65] and larger estuaries has been well documented. Studies in Florida show spawning in deep waters inshore, from November to April, with climaxes in February and March [66, 67].

### Trophic ecology

*Pogonias courbina* feeds largely on benthic invertebrates, mainly bivalves and crabs in Mar Chiquita coastal lagoon. The crab *Cyrtograpsus angulatus* and the bivalve *Brachidontes rodriguezi* were the main prey items of *P*. *courbina* [68]. The diet composition changes between seasons. This seasonal feeding pattern reorients its foraging strategy and trophic niche breadth.

*Pogonias courbina* is well adapted for bottom feeding. This species has a subterminal, nearly horizontal mouth [53] with small villiform teeth, arranged in an irregular way, decreasing in size backwards [69]. Vomer, palatine, and tongue have no teeth, but instead strong pharyngeal teeth crush the shells of mollusks and crabs before swallowing. In shallow waters, black drums fed on the bottom in a vertical position so that their tails stick out of the water (ALC pers. observ. in aquarium).
